# Brain-Computer Interface: Advancement and Challenges

**DOI:** 10.3390/s21175746

**Published:** 2021-08-26

**Authors:** M. F. Mridha, Sujoy Chandra Das, Muhammad Mohsin Kabir, Aklima Akter Lima, Md. Rashedul Islam, Yutaka Watanobe

**Affiliations:** 1 Department of Computer Science and Engineering, Bangladesh University of Business and Technology, Dhaka 1216, Bangladesh; firoz@bubt.edu.bd (M.F.M.); dsujoy.cse@gmail.com (S.C.D.); mdmkabi@gmail.com (M.M.K.); hossain.limuu@gmail.com (A.A.L.); 2 Department of Computer Science and Engineering, University of Asia Pacific, Dhaka 1216, Bangladesh; 3 Department of Computer Science and Engineering, University of Aizu, Aizu-Wakamatsu 965-8580, Japan; yutaka@u-aizu.ac.jp

**Keywords:** brain-computer interface, signal processing, biomedical sensors, systematic review

## Abstract

Brain-Computer Interface (BCI) is an advanced and multidisciplinary active research domain based on neuroscience, signal processing, biomedical sensors, hardware, etc. Since the last decades, several groundbreaking research has been conducted in this domain. Still, no comprehensive review that covers the BCI domain completely has been conducted yet. Hence, a comprehensive overview of the BCI domain is presented in this study. This study covers several applications of BCI and upholds the significance of this domain. Then, each element of BCI systems, including techniques, datasets, feature extraction methods, evaluation measurement matrices, existing BCI algorithms, and classifiers, are explained concisely. In addition, a brief overview of the technologies or hardware, mostly sensors used in BCI, is appended. Finally, the paper investigates several unsolved challenges of the BCI and explains them with possible solutions.

## 1. Introduction

The quest for direct communication between a person and a computer has always been an attractive topic for scientists and researchers. The Brain-Computer Interface (BCI) system has directly connected the human brain and the outside environment. The BCI is a real-time brain-machine interface that interacts with external parameters. The BCI system employs the user’s brain activity signals as a medium for communication between the person and the computer, translated into the required output. It enables users to operate external devices that are not controlled by peripheral nerves or muscles via brain activity.

BCI has always been a fascinating domain for researchers. Recently, it has become a charming area of scientific inquiry and has become a possible means of proving a direct connection between the brain and technology. Many research and development projects have implemented this concept, and it has also become one of the fastest expanding fields of scientific inquiry. Many scientists tried and applied various communication methods between humans and computers in different BCI forms. However, it has progressed from a simple concept in the early days of digital technology to extremely complex signal recognition, recording, and analysis techniques today. In 1929, Hans Berger [1] became the first person to record an Electroencephalogram (EEG) [2], which shows the electrical activity of the brain that is measured through the scalp of a human brain. The author tried it on a boy with a brain tumor; since then, EEG signals have been used clinically to identify brain disorders. Vidal [3] made the first effort to communicate between a human and a computer using EEG in 1973, coining the phrase “Brain-Computer Interface”. The author listed all of the components required to construct a functional BCI. He made an experiment room that was separated from the control and computer rooms. In the experiment room, three screens were required; the subject’s EEG was to be sent to an amplifier the size of an entire desk in the control area, including two more screens and a printer.

The concept of combining brains and technology has constantly stimulated people’s interest, and it has become a reality because of recent advancements in neurology and engineering, which have opened the pathway to repairing and possibly enhancing human physical and mental capacities. The sector flourishing the most based on BCI is considered the medical application sector. Cochlear implants [4] for the deaf and deep brain stimulation for Parkinson’s illness are examples of medical uses becoming more prevalent. In addition to these medical applications, security, lie detection, alertness monitoring, telepresence, gaming, education, art, and human enhancement are just a few uses for brain–computer interfaces (BCIs), also known as brain–machine interfaces or BMIs [5]. Every application based on BCI follows different approaches and methods. Each method has its own set of benefits and drawbacks. The degree to which a performance can be enhanced while minute-to-minute and day-to-day volatility are reduced is crucial for the future of BCI technology. Such advancements rely on the capacity to systematically evaluate and contrast different BCI techniques, allowing for the most promising approaches to be discovered. In addition, this versatility around BCI technologies in different sectors and their applications can seem so complex yet so structured. Most of the BCI applications follow a standard structure and system. This basic structure of BCI consists of signal acquisition, pre-processing, feature extraction, classification, and control of the devices. The signal acquisition paves the way to connecting a brain and a computer and to gathering knowledge from signals. The three parts of pre-processing, feature extraction, and classification are responsible for making the associated signal more usable. Lastly, control of the devices points out the primary motivation: to use the signals in an application, prosthetic, etc.

The outstanding compatibility of various methods and procedures in BCI systems demands extensive research. A few research studies on specific features of BCI have also been conducted. Given all of the excellent BCI research, a comprehensive survey is now necessary. Therefore, an extensive survey analysis was attempted and focused on nine review papers featured in this study. Most surveys, however, do not address contemporary trends and application as well as the purpose and limits of BCI methods. Now, an overview and comparisons of the known reviews of the literature on BCI are shown in Table 1.

Abiri, R. et al. [6] evaluated the current review on EEG-based various experimental paradigms used by BCI systems. For each experimental paradigm, the researchers experimented with different EEG decoding algorithms and classification methods. The researchers overviewed the paradigms such as Motor imagery paradigms, Body kinematics, Visual P300, Evoked potential, and Error related potential and the hybrid paradigms analyzed with the classification methods and their applications. Researchers have already faced some severe issues while exploring BCI paradigms, including training time and fatigue, signal processing, and novel decoders; shared control to supervisory control in closed-loop; etc. Tiwari, N. et al. [7] provided a complete assessment of the evolution of BCI and a fundamental introduction to brain functioning. An extensive comprehensive revision of the anatomy of the human brain, BCI, and its phases; the methods for extracting signals; and the algorithms for putting the extracted information to use was offered. The authors explained the steps of BCI, which consisted of signal acquisition, feature extraction, and signal classification. As the human brain is complex, human-generated thoughts are non-stationary, and generated signals are nonlinear. Thus, the challenging aspect is to develop a system to find deeper insights from the human brain; then, BCI application will perform better with these deeper insights. Vasiljevic, G.A.M. et al. [8] presented a Systematic Literature Review (SLR) conclusion of BCI games employing consumer-grade gadgets. The authors analyzed the collected data to provide a comprehensive picture of the existing reality and obstacles for HCI of BCI-based games utilizing consumer-grade equipment. According to the observations, numerous games with more straightforward commands were designed for research objectives, and there was a growing amount of more user-friendly BCI games, particularly for recreation. However, this study is limited to the process of search and classification. Martini, M.L. et al. [9] investigated existing BCI sensory modalities to convey perspectives as technology improves. The sensor element of a BCI circuit determines the quality of brain pattern recognition, and numerous sensor modalities are presently used for system applications, which are generally either electrode-based or functional neuroimaging-based. Sensors differed significantly in their inherent spatial and temporal capabilities along with practical considerations such as invasiveness, mobility, and maintenance. Bablani, A. et al. [10] examined brain reactions utilizing invasive and noninvasive acquisition techniques, which included electrocorticography (ECoG), electroencephalography (EEG), magnetoencephalography (MEG), and magnetic resonance imaging (MRI). For operating any application, such responses must be interpreted utilizing machine learning and pattern recognition technologies. A short analysis of the existing feature extraction techniques and classification algorithms applicable to brain data has been presented in this study.

Fleury, M. et al. [11] described various haptic interface paradigms, including SMR, P300, and SSSEP, and approaches for designing relevant haptic systems. The researchers found significant trends in utilizing haptics in BCIs and NF and evaluated various solutions. Haptic interfaces could improve productivity and could improve the relevance of feedback delivered, especially in motor restoration using the SMR paradigm. Torres, E.P. et al. [12] conducted an overview of relevant research literature from 2015 to 2020. It provides trends and a comparison of methods used in new implementations from a BCI perspective. An explanation of datasets, emotion elicitation methods, feature extraction and selection, classification algorithms, and performance evaluation is presented. Zhang, X. et al. [13] discussed the classification of noninvasive brain signals and the fundamentals of deep learning algorithms. This study significantly gives an overview of brain signals and deep learning approaches to enable users to understand BCI research. The prominent deep learning techniques and cutting-edge models for brain signals are presented in this paper, together with specific ideas for selecting the best deep learning models. Gu, X. et al. [14] investigated the most current research on EEG signal detection technologies and computational intelligence methodologies in BCI systems that filled in the loopholes in the five-year systematic review (2015–2019). The authors demonstrated sophisticated signal detecting and augmentation technologies for collecting and cleaning EEG signals. The researchers also exhibited computational intelligence techniques, such as interpretable fuzzy models, transfer learning, deep learning, and combinations for monitoring, maintaining, or tracking human cognitive states and the results of operations in typical applications.

**Figure 1:**
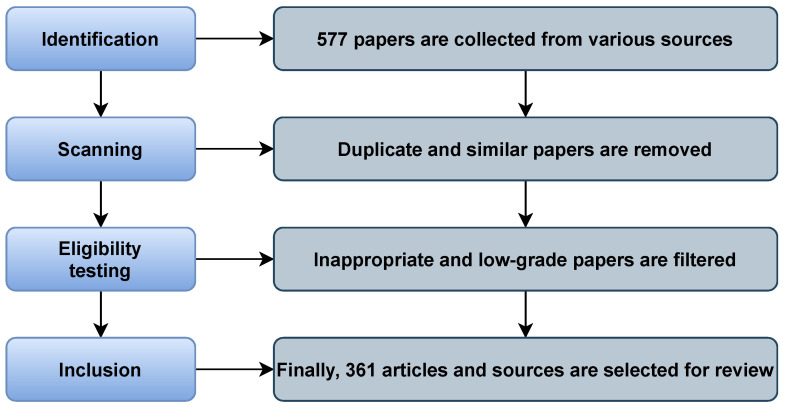
The PRISMA process that is followed in this article.

The study necessitated a compendium of scholarly studies covering 1970 to 2021 since we analyze BCI in detail in this literature review. We specialized in the empirical literature on BCI from 2000 to 2021. For historical purposes, such as the invention of BCI systems and their techniques, we selected some publications before 2000. Kitchenham [15,16] established the Systematic Literature Review (SLR) method, which is applied in the research and comprises three phases: organizing, executing, and documenting the review. The SLR methodologies attempted to address all possible questions that could arise as the current research progresses. The recent study’s purpose is to examine the findings of numerous key research areas. The PRISMA (Preferred Reporting Items for Systematic Reviews and Meta-Analyses) guidelines were used to put together the essential materials for this study, which consists of four parts: identification, scanning, eligibility testing, and inclusion. We gathered 577 papers from a variety of sources and weeded out duplicates and similar articles. Finally, we carefully chose 361 articles and sources for monitoring and review. The PRISMA process is presented in Figure 1.

However, this research looks at the present challenges and difficulties in this BCI field. Furthermore, this study generates ideas and suggestions for future research subjects. The following are the research’s total contributions:

- The paper explicitly illustrates Brain-Computer Interface’s (BCI) present, past, and future trends and technologies.
- The paper presents a taxonomy of BCI and elaborates on the few traditional BCI systems with workflow and architectural concepts.
- The paper investigates some BCI tools and datasets. The datasets are also classified on different BCI research domains.
- In addition, the paper demonstrates the application of BCI, explores a few unsolved challenges, and analyzes the opportunities.

After reading this section, one should understand BCI and how to get started with it. Our motivation to work with BCI started from a desire to learn more about this domain. Furthermore, the BCI has a bright future ahead of it, as it has a lot to offer in the medical field and in everyday life. BCI can change one’s incapability and can make life and work easy, as detailed in the following section. The applications, problems, future, and social consequences of BCI have also fueled our enthusiasm for this research.

The remainder of the paper is constructed as follows. The motivation of this work and diverse applications of BCI systems are illustrated in Section 2. Section 3 describes the structure of BCI and briefly reviews the most popular techniques of BCI. In Section 5, different categories of datasets available publicly are displayed. In Section 7, the most widely used methods for signal enhancement and feature extraction of BCI are discussed. The most commonly known classifiers are reviewed in Section 8. A broad discussion on the evaluation metrics for BCI is given in Section 9. The challenges faced most commonly during the BCI process are reviewed in Section 10. Lastly, this paper provides a conclusion in Section 11.

## 2. Applications of BCI

BCIs may be used for various purposes and the application determines the design of a BCI. According to Nijholt [17], applications based on BCI have two methods of usability. One can command whether the other one can be observed or monitored. The majority of command applications concentrate on manipulating brain impulses using electrodes to control an external device. On the other hand, applications that involve observation focus on recognizing a subject’s mental and emotional state to behave appropriately depending on their surroundings. Some applications of BCI [18] based on usability are described below:

### 2.1. Biomedical Applications

The majority of BCI integrations and research have been focused on medical applications, with many BCIs aiming to replace or restore Central Nervous System (CNS) functioning lost with sickness or by accident. Other BCIs are more narrowly targeted. In diagnostic applications, on treatment and motor rehabilitation following CNS disease or trauma, BCIs for biological purposes are also employed in affective application domains. Biomedical technologies and applications can minimize extended periods of sickness, can provide supervision and protection by empowering persons with mobility difficulties, and can support their rehabilitation. The necessity to build accurate technology that can cope with potentially abnormal brain responses that might occur due to diseases such as brain stroke is a significant challenge in developing such platforms [19]. The following subsections go through each of these applications in further detail.

#### 2.1.1. Substitute to CNS

These substitution means that it can repair or replace CNS functioning lost due to diseases such as paralysis and spinal cord injury due to stroke or trauma. In addition, due to changed brain functions, individuals with such illnesses might suffer and developing such technology can be difficult. Myoelectrics, known as a motor action potential, which captures electrical impulses in muscles, is now used in several robotic prosthetics. Bousseta, R. et al. [20] provided an experimental technology for controlling the movement of a robotic prosthetic arm with mental imagery and using cognitive tasks, which can move in four directions like left, right, up, and down.

#### 2.1.2. Assessment and Diagnosis

The usage of BCIs in a clinical context can also help with assessment and diagnosis. Perales [21] suggested a BCI for assessing the attention of youngsters with cerebral palsy while playing games. Another research [22] looked into using BCI to capture EEG characteristics as a tool for diagnosing schizophrenia. There are also various diagnostic methods such as the detection of brain tumors [23], the identification of breast cancer [24], parkinson’s disease [25] etc. Diagnoses of several diseases in children including epilepsy, neurodegenerative disorders, motor disabilities, inattentiveness, or different types of ADHD [26] are possible. Assessment and diagnosis technologies are essential to patient well-being. Their functioning must be fine-tuned to guarantee that they are safe, acceptable, and accurate to industry standards.

#### 2.1.3. Therapy or Rehabilitation

BCI is being used in therapeutic applications besides neurological application and prosthetics nowadays. Among the many applications, post-stroke motor rehabilitation shows promising results using BCI. Stroke is a disease that causes long-term disability to the human body and hampers all kinds of motor or vigorous activity due to an impediment of blood flow. Stroke rehabilitation application has promised to aid these activities or user imaginations through a robot or other types of machinery [27,28,29]. Some other applications treat neurological disorders such as Parkinson’s disease (PD), cluster headaches, tinnitus, etc. Deep Brain Stimulation (DBS) is an established treatment for PD as it delivers electrical impulses to a targeted area of the brain responsible for the symptoms [30]. Some stimulation BCI devices are used to process calmness during migraine attacks and cluster headaches. Lastly, a CNS disorder known as tinnitus is also in development to provide treatment by identifying brain patterns that are changed due to the disease [31]. Lastly, treatment for auditory verbal hallucinations (AVHs), best known as schizophrenia, is a possibility besides diagnosis [32,33].

#### 2.1.4. Affective Computing

Users’ emotions and state of mind are observed in affective computing BCIs, with the possibility of altering their surrounding environment to improve or change that emotion. Ehrlich, S. et al. [34] created a closed-loop system in which music is generated and then replayed to listeners based on their emotional state. Human emotional states and sensory connections can be studied with a device that is related to BCI system. Patients suffering neurological diseases also can benefit from affective computing to help them convey their feelings to others [35].

### 2.2. Non-Biomedical Applications

BCI technologies have shown economic promise in recent years, notably in the field of non-biomedical applications. Most of these applications consist of entertaining applications, games, and emotional computation. In comparison, researchers focus on robustness and high efficiency in medical and military applications, and innovations targeted at leisure or lifestyle demand a greater emphasis on enjoyment and social elements. The most challenging aspect of this entertainment application is that it must be a user favorite to be commercially successful. As an example, some of the most popular forms of amusement are as follows:

#### 2.2.1. Gaming

BCIs focused mainly on the gaming sector have grown in importance as a research topic. However, gaming BCIs are currently a poor substitute for standard game control methods [36]. BCI in gaming is an area where further research is needed to make games more user-friendly. In some cases, EEG data make BCI games more utilizable and increase engagement, and the system tracks each player’s enthusiasm level and activates dynamic difficulty adjustment (DDA) when the players’ excitement drops [37]. When developing such systems, fine-tuning the algorithms that regulate the game’s behavior is a big challenge. Some other games are based on BCI, as it is not visually intense and the graphics are not compatible with the recent generation. With setbacks, there is an engaging future for an Adaptation of P300 based Brain-Computer Interface for Gaming [38], which is gaining more popularity as these are very flexible to play.

#### 2.2.2. Industry

EEG-based BCIs can also be used in industrial robotics, increasing worker safety by keeping people away from potentially demanding jobs. These technologies could substitute the time-consuming button and joystick systems used to teach robots in industrial applications; can detect when a person is too tired or ill to operate the machinery; and can take the necessary precautions to avoid injury, such as stopping the machinery [38].

#### 2.2.3. Artistic Application

The four types of artistic applications recognized by BCIs are passive, selective, direct, and collaborative. Passive artistic BCIs need not require active user input to use the user’s brain activity to determine which pre-programmed responses to produce. Every user has had some limited control over the process within selective systems. Still, they will never be in charge of the creative product. Direct artistic BCIs provide users with far more flexibility, generally allowing them to choose items from extensive menus, such as brush type and managing brush stroke movements [39]. Lastly, the collaborative system is controlled by different users [40].

#### 2.2.4. Transport

BCI is used in transportation monitoring which tracks awareness to assess driver weariness and to enhance airline pilot performances. In the BCI system, mistakes can be costly regarding lives and monetary obligations on the entities involved when such technologies are utilized in critical applications [41,42].

## 3. Structure of BCI

**Figure 2:**
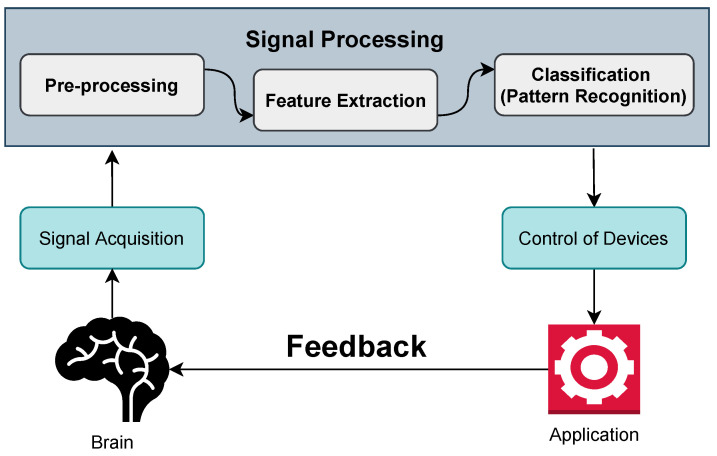
Basic architecture of a BCI system.

The BCI system operates with a closed-loop system. Every action taken by the user is met with some feedback. For example, an imagined hand movement might result in a command that causes a robotic arm to move. This simple movement of this arm needs a lot of processes inside it. It starts from the brain, which is one of our body’s most extensive and most complicated organs. It is made up of billions of nerves that link billions of synapses to communicate. The processes from taking signals from the human brain to transforming into a workable command are shown in Figure 2 and described below:

- Signal acquisition: In the case of BCI, it is a process of taking samples of signals that measure the brain activity and turning them into commands that can control a virtual or real-world application. The various techniques of BCI for signal acquisition are described later.
- Pre-processing: After the signal acquisition, the pre-processing of signals is needed. In most cases, the collected signals from the brain are noisy and impaired with artifacts. This step helps to clean this noise and artifacts with different methods and filtering. That is why it is named signal enhancement.
- Feature extraction: The next stage is feature extraction, which involves analyzing the signal and extracting data. As the brain activity signal is complicated, it is hard to extract useful information just by analyzing it. It is thus necessary to employ processing algorithms that enable the extraction of features of a brain, such as a person’s purpose.
- Classification: The next step is to apply classification techniques to the signal, free of artifacts. The classification aids in determining the type of mental task the person is performing or the person’s command.
- Control of devices: The classification step sends a command to the feedback device or application. It may be a computer, for example, where the signal is used to move a cursor, or a robotic arm, where the signal is utilized to move the arm.

The basic architecture of the BCI system was explained in the preceding section. It prompts us to investigate the classification of BCI system. Based upon various techniques, BCI system is classified. The BCI techniques are discussed in following parts.

**Figure 3:**
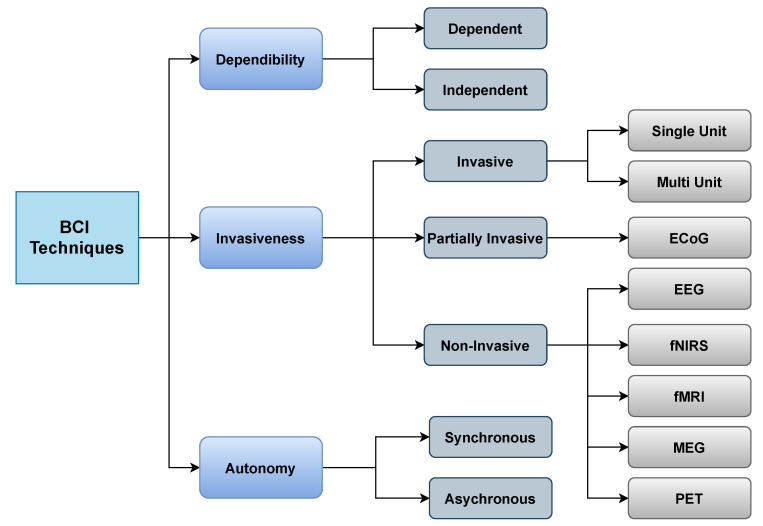
The classification/taxonomy of the BCI system.

From the above Figure 3, we can classify BCI from different aspects such as dependability, invasiveness, and autonomy.

- Dependability: BCI can be classified as dependent or independent. Dependent BCIs necessitate certain types of motor control from the operator or healthy subjects, such as gaze control. On the other hand, independent BCIs do not enable the individual to exert any form of motor control; this type of BCI is appropriate for stroke patients or seriously disabled patients.
- Invasiveness: BCI is also classified into three types according to invasiveness: invasive, partially invasive, and non-invasive. Invasive BCIs are by far the most accurate as they are implanted directly into the cortex, allowing researchers to monitor the activity of every neuron. Invasive varieties of BCI are inserted directly into the brain throughout neurosurgery. There are two types of invasive BCIs: single unit BCIs, which detect signals from a single place of brain cells, and multi-unit BCIs, which detect signals from several areas. Semi-invasive BCIs use Electrocorticography (ECoG), a kind of signal platform that enables electrodes to be placed on the attainable edge of the brain to detect electrical impulses originating from the cerebral cortex. Although this procedure is less intrusive, it still necessitates a surgical opening in the brain. Noninvasive BCIs use external sensing rather than brain implants. Electroencephalography (EEG), Magnetoencephalography (MEG), Positron emission tomography (PET), Functional magnetic resonance imaging (fMRI), and Functional near-infrared spectroscopy (fNIRS) are all noninvasive techniques used it to analyze the brain. However, because of the low cost and portability of the gear, EEG is the most commonly used.
- Autonomy: BCI can operate either in a synchronous or asynchronous manner. Time-dependent or time-independent interactions between the user and system are possible. The system is known as synchronous BCI if the interaction is carried out within a particular amount of time in response to a cue supplied by the system. In asynchronous BCI, the subject can create a mental task at a certain time to engage with the system. Synchronous BCIs are less user-friendly than asynchronous BCIs; however, designing one is substantially easier than developing an asynchronous BCI.

As the motive of this research work is to focus on advancements of BCI, the most advanced and mostly used techniques that is based on invasiveness are described in the following part. Based on invasiveness, BCI is classified into three categories that are more familiar. In the consequent section, we address these three categories and describe them elaborately.

### 3.1. Invasive

The types of BCI that are invasive are inserted directly into the brain with neurosurgery. Invasive BCIs seem to be the most accurate even though they are implanted directly into the cortex as it is allowed to track every neuron’s action. Invasive BCI also has two units rather than parts. The first unit is single-unit BCIs that detect signals from a single location of brain cells, whereas multi-unit BCIs detect numerous areas, the second unit [43]. However, the neurosurgery treatment has various flaws, such as the possibility of scar tissue formation. The body responds to the foreign object by forming a scar around the electrodes, leading the signal to deteriorate. Since neurosurgery is a dangerous and costly procedure, invasive BCI is mainly used on blind and paralyzed patients.

### 3.2. Partially Invasive

Although this approach is not as intrusive, it still involves brain surgery. Electrocorticography (ECoG) is a sort of partially invasive BCI monitoring system that places electrodes in the cortex surface of the brain to produce signals with electrical activity. For example, blinking allows your brain to discharge electrical activity. When investigating signals, though, these involuntary actions are generally not of interest since they are in the way of what we search for. It is a form of noise. ECoGs are less considered with noise than non-invasive BCI, making interpretation easier [44].

#### Electrocorticography (ECoG)

Electrocorticography (ECoG) [45] is an partially invasive method that measures the brain’s electrical activity. In another sense, the participant’s skull must be evacuated, and the electrodes must be placed right at the brain’s service. Consequently, this electrode is located on the skull. The particular resolution of the recorded signals is considerably better than EEG. The signal-to-noise ratio is superior compared with the closer proximity to cerebral activity. Furthermore, motion artifacts such as blinks and eye movement have a significantly lower impact on ECoG signals. However, ECoG would only be helpful in the accessible brain area and is close to impossible to utilize outside of a surgical setting [46].

### 3.3. Noninvasive

Noninvasive neuroimaging technologies have also been used as interfaces in human research. Noninvasive EEG-based BCIs account for the vast bulk of published BCI research. EEG-based noninvasive technologies and interfaces have been employed in a considerably more comprehensive range of applications. Noninvasive apps and technologies are becoming increasingly popular in recent years since they do not require any brain surgery. In the noninvasive mode, a headpiece or helmet-like electrode is utilized outside the skull to measure the signal by causing electrical activity in the brain. There are some well-known and widely used ways for measuring these electrical activity or potentials, such as Electroencephalography (EEG), Magnetoencephalography (MEG), Functional Magnetic Resonance Imaging (fMRI), Facial Near Infrared Spectroscopy (fNIRS), and Positron Emission Tomography (PET). An elaborate description of BCI techniques is given below:

#### 3.3.1. Electroencephalography (EEG)

EG monitors electrical activity in the scalp generated by activating a few of the brain’s neurons. Several electrodes implanted on the scalp directly, mainly on the cortex, are often used to record these electrical activities quickly. For its excellent temporal resolution, ease of use, safety, and affordability, EEG is the most used technology for capturing brain activity. Active electrodes and passive electrodes are indeed the two types of electrodes that can be utilized. Active electrodes usually feature an integrated amplifier, whereas passive electrodes require an external amplifier to magnify the detected signals. The prime objective of implementing either embedded or external amplifiers is to lessen the impact of background noise and other signal weaknesses caused by cable movement. One of the issues with EEG is that it necessitates the use of gel or saline solutions to lower the resistance of skin-electrode contact. Furthermore, the signal quality is poor, and it is altered by background noise. The International 10–20 system [47] is often used to implant electrodes over the scalp surface for recording purposes. The electrical activities across various frequency bands are used to describe EEG in general.

#### 3.3.2. Magnetoencephalography (MEG)

The magnetic fields created by current flow in the brain are measured using MEG (Magnetoencephalography). Electric fields have significantly more interrupted travel via the skull than magnetic fields, therefore it has superior spatial resolution than EEG. A functional neuroimaging technique is applied to measure and evaluate the brain’s magnetic field. MEG operates on the outside of the head and is now a part of the clinical treatment regularly. David Choen [48,49] was the first to invent it in 1968 by utilizing a conduction copper detector inside a shielded chamber to reduce background noise. Improved MEG signals have recently been produced using more sensitive sensors such as superconducting quantum interference devices (SQUID) [50]. MEG has become significant, especially for patients with epilepsy and brain tumors. It may aid in detecting regions of the brain with average function in individuals with epilepsy, tumors, or other mass lesions. MEG operates with magnetic waves rather than electrical waves so that it could contribute additional information to EEG. MEG is also capable of capturing signals with high temporal and spatial resolution. Therefore, to detect cerebral activity that creates tiny magnetic fields the scanners must be closer to the brain’s surface. As a result, specific sensors are required for MEG, such as superconducting quantum interference (SQUID) sensors [51].

#### 3.3.3. Functional Magnetic Resonance Imaging (fMRI)

Noninvasive functional magnetic resonance imaging (fMRI) is used to evaluate the fluctuation in blood oxygen levels throughout brain activities. fMRI has an excellent spatial resolution, which makes it ideal for identifying active areas of the brain [52]. The time resolution of fMRI is comparatively low, ranging from 1 to 2 s [53]. It also has low resolution when it comes to head movements, which could result in artifacts. In the 1990s, functional magnetic resonance imaging (fMRI) was created. It is a noninvasive and safe technology that does not include the use of radiation, is simple to use, and has great spatial and temporal resolution. Hemoglobin in capillary red blood cells in the brain transports oxygen to the neurons. As a result of the increased demand for oxygen, blood flow increases. If haemoglobin is oxygenated, its magnetic properties vary. The MRI equipment, which is a cylindrical tube with a strong electromagnet, can determine which regions of the brain are activated because of this difference. That is how fMRI works. There is also a specific application or software known as diffusion MRI, which generates images from the data or results that use water molecules’ diffusion. Diffusion-weighted and diffusion tensor imaging (DWI/DTI) facilitates this exploration of the microarchitecture of the brain. Diffusion-weighted magnetic resonance imaging (DWI or DW-MRI) imaging renders picture variation depending on variances in the degree of diffusion of water particles inside the brain. Diffusion depicts the stochastic thermic mobility of particles. Diffusion inside the brain is defined by several agents, including representing particles beneath study, the temperature, and the microenvironmental structure in which the diffusion occurs [54]. Diffusion tensor imaging (DTI) investigates the three-dimensional form of the diffusion, also recognized as diffusion tensor. It is a powerful MRI modality that produces directional knowledge about the water motion in a voxel. It exhibits noninvasively microscopic tissue features that surpass the ability of any other imaging methods [55].

#### 3.3.4. Functional Near-Infrared Spectroscopy (fNIRS)

The infrared radiation is projected into the brain using fNIRS equipment [53,56] to monitor improvements in specific wavelengths as the light is reflected. fNIRS often detects changes in regional blood volume and oxygenation. When a particular area of the brain works, it requires additional oxygen, which is given to the neurons via capillary red blood cells—the increased blood flow in the brain areas that would be most active at a given time. fMRI is a technique that monitors variations in oxygen levels caused by various activities. As a result, images with a high spatial resolution (1 cm) but lower temporal resolution (>2–5 s) could be obtained, comparable with standard functional magnetic resonance imaging.

#### 3.3.5. Positron Emission Tomography (PET)

PET (positron emission tomography) is a sophisticated imaging tool for examining brain activities in real-time. It enables noninvasive measurement of cerebral blood flow, metabolism, and receptor binding in the brain. Due to the relatively high prices and complexity of the accompanying infrastructure, including cyclotrons, PET scanners, and radio chemistry laboratories, PET was previously only used in research. PET has been widely employed in clinical neurology in recent years due to technological improvements and the proliferation of PET scanners to better our understanding of disease etiology, to help in diagnosis, and to monitor disease progression and response to therapy [57]. PET medications such as radiolabeled choline, fluciclovine (18F-FACBC), and compounds targeting prostate-specific membrane antigen are now being researched and explored to improve noninvasive prostate cancer localization diagnostic performance [58].

## 4. Brain Control Signals

**Figure 4:**
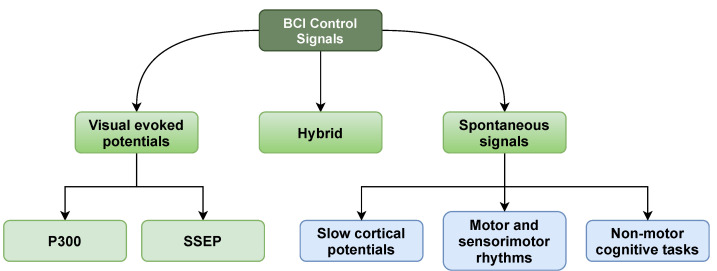
The basic architecture of BCI control signals.

The brain-computer interface (BCI) is based on signal amplification that comes directly from the brain. Several of these signals are simple to extract, while others are more difficult and require additional preprocessing [53]. These control signals can be classified into one of three groups: (1) evoked signals, (2) spontaneous signals, and (3) hybrid signals. A detailed overview of the three categories is given below. The control signals classification is shown in Figure 4.

### 4.1. Visual Evoked Potentials

Electrical potentials evoked by short visual stimuli are known as VEPs. The visual cortex’s potentials are monitored, and the waveforms are derived from the EEG. VEPs are generally used to assess the visual pathways from the eye to the brain’s visual cortex. Middendorf et al. published a procedure for measuring the position of the user’s gaze using VEPs in 2000 [59]. The user is confronted with a screen that displays several virtual buttons that flash at varied rates. The frequency of the photic driving reflex over the user’s visual brain is determined after the user focuses their gaze on a button. Whenever the frequency of a shown button equals the frequency of the user, the system concludes that the user wants to pick it. Steady-State Evoked Potentials (SSEP) and P300 are two of the most well-evoked signals. External stimulation is required for evoked signals that can be unpleasant, awkward, and exhausting for the individual.

#### 4.1.1. Steady-State Evoked Potential (SSEP)

SSEP signals are produced when a patient experiences periodic stimuli such as a flickering picture, modulated sound, or even vibrations [60,61]. The strength of the EEG signal in the brain must grow to meet the stimulus frequency. Signals in many brain locations are observed in terms of the sensory process. SSEP signals of different forms, such as steady-state visual potentials (SSVEPs), somatosensory SSEP, and auditory SSEP, are found. SSVEP is widely used in a variety of applications. These are normal brain reactions to repeating stimuli, which vary depending on the frequency with which they are presented. Although there are instances of BCI paradigms utilizing somatosensory (SSSEP) or auditory (SSAEP) stimuli, they are generally induced using visual stimuli (steady-state visually evoked potentials, SSVEP) [62].

#### 4.1.2. P300 Evoked Potentials (P300)

The peaks in an EEG generated by infrequent visual, auditory, or somatosensory inputs are known as P300 evoked potentials. Without the need for training to use P300-based BCI systems. A matrix of symbols, in which selection is dependent on the participant’s gaze, is a prominent use of P300-based BCI systems. Such a signal is typically produced using an “odd-ball” paradigm. The user is asked to respond to a random succession of stimuli, which is less frequent than others [63]. The P300-based EEG waves are triggered when this unusual stimulus is significant to the person. P300 does not reasonably require any subject training, although, it does need repetitive stimulation, which may tire the subject and may cause inconsistencies.

### 4.2. Spontaneous Signals

With no external cues, the person produces random signals willingly. These signals are produced without any external stimuli (somatosensory, aural, or visual). Motor and sensorimotor rhythms, Slow Cortical Potentials (SCPs), and non-motor cognitive signals are some of the most prominent spontaneous signals [53].

#### 4.2.1. Motor and Sensorimotor Rhythms

Motor activities are linked to motor and sensorimotor rhythms. Sensorimotor rhythms are rhythmic oscillations in electrophysiological brain activity in the mu (Rolandic band, 7–13 Hz) and beta (13–30 Hz) frequencies. Motor imagery is the process of converting a participant’s motor intentions into control signals employing motor imagery conditions [64]. The left-hand motion, in an instance, may result in EEG signals in the and rhythms and a decrease in certain motor cortex areas (8–12 Hz) and (18–26 Hz). Depending on the motor imagery rhythms, various applications can be used such as controlling a mouse or playing a game.

#### 4.2.2. Slow Cortical Potentials (SCP)

SCP is indeed an EEG signal with a frequency less than 1 Hz [65]. It is a low-frequency potential observed in the frontal and central portions of the cortex and depolarization level variations throughout the cortical dendrites. SCP is a highly gradual change in brain activity, either positive or negative, that can only last milliseconds to several seconds. Through operant conditioning, the subject can control the movement of such signals. As a result, extensive training may be required in addition to that needed for motor rhythms. Many studies no longer choose SCP, and motor and sensorimotor rhythms have taken their place.

#### 4.2.3. Non-Motor Cognitive Tasks

Cognitive objectives are utilized to drive the BCI in non-motor cognitive tasks. Several tasks, such as musical imagination, visual counting, mental rotation, and mathematical computation, might be completed [66]. Penny, W.D. et al. [67] used a pattern classifier with unclear parameters. The individual performed simple subtraction in one of their non-motor cognitive activities.

### 4.3. Hybrid Signals

The term “hybrid signals” refers to the utilization of a mixture of brain-generated signals for control. As a result, instead of measuring and using only one signal in the BCI system, a mix of signals is used. The fundamental goal of using two or more types of brain signals as input to a BCI system is to increase dependability while avoiding the drawbacks of each signal type [68].

Some research is addressed that the types of brain signals are classified into two categories [10]. These are event-related potentials and evoked brain potential. Three varieties are organized for evoked brain potential: Visual Evoked Potential (VEP), Tactile Evoked Potential (TEP), and Auditory Evoked Potential (AEP) [69].

## 5. Dataset

While analyzing the literature on BCI systems, we discovered various often used datasets that researchers used while implementing these techniques. In terms of the research, EEG is now the most frequent method for collecting brain data in BCI. As this is a noninvasive method and has convenient handling for most datasets, an EEG signal is used. However, for a variety of reasons, EEG does not provide a comprehensive method of data collection. It needs a variety of fixed things to acquire the data. Firstly, the signal must be acquired and stored by some subject, participants, or patients. It is unsuitable when only one subject requires the same arrangement as multiple subjects to obtain data. After the subjects are prepared, the electrodes (a gear mounted on the scalp) are attached to the individuals to capture and measure data. This data collection method lasted for several sessions, with a particular recording period determined by the work’s purpose. The saved data in these sessions and recordings are primarily brain signals measured by a brain’s action on a sure thing, such as a video or a picture. EEG signals differ from one participant to the next and from one session to the next. In this section, the datasets as well as the subjects and electrodes, channels, and sessions are described. The explanation is tabulated in Table 2, Table 3, Table 4, Table 5, Table 6, Table 7 and Table 8. In Table 2, some popular motor imagery datasets are illustrated. The most beneficial option for creating BCIs is motor imagery (MI) impulses captured via EEG, which offers a great degree of mobility. It enables people with motor disabilities to communicate with the device by envisioning motor movements without any external stimuli generated from the motor cortex. A few datasets based on error-related potentials (ErrPs) are exhibited in Table 3. ErrPs is an EEG dataset that utilizes a P300-based BCI speller to boost the performance of BCIs. Detecting and fixing errors of the neuronal signature of a user’s knowledge linked to a brain pattern is known as error-related potentials (ErrPs). Affective computing improves human–machine communication by identifying human emotions. Some mostly used emotion recognition datasets are shown in Table 4. Various EEG-based BCI devices can detect the user’s emotional states to make contact effortless, more useable, and practical. The emotions extracted in emotion-recognition datasets are valence, arousal, calm, positive, exciting, happy, sad, neutral, and fear. In addition, it is certainly clear by now that brain signals or memory are a mixed emotion. The part where all of these mixed emotions are gathered from different body parts is known as a miscellaneous part of the brain. Therefore, miscellaneous datasets include memory signals, brain images, brain signals, etc. Some miscellaneous datasets are represented in Table 5. In EEG-based BCI, the signals can detect eye movement such as eye blinks, eye states, etc. The BCI datasets of eye blinks or movements include voluntary and involuntary eye states, blinks, and activities are illustrated in Table 6. Subsequently, the electrical response in the brain to a specific motor or cognitive event such as a stimulus is known as an event-related potential (ERP). An unwanted sound, a sparking light, or a blinking eye can be an example of a stimulus. BCI utilizing ERPs attempts to track attention, weariness, and the brain’s reaction to this event-related stimulus. Table 7 is encapsulated with popular ERP datasets around. Moreover, the visual information-processing mechanism in the brain is reflected in Visually Evoked Potentials (VEPs). Flashing objects in the form of shifting colors or a reversing grid are frequent visual stimulators. The CRT/LCD monitor or flash tube/infrared diode (LED) is utilized for stimulus display in VEP-based BCIs. Frequently used VEP-based datasets with these utilized objects are represented in Table 8.

However, the dataset covers information recorded from the beginning of BCI. To extract information from datasets, feature extraction methods are necessary, which is reviewed in the following section.

## 6. Signal Preprocessing and Signal Enhancement

In most situations, the signal or data measured or extracted from datasets are filled with noise. With a natural human activity such as eye blinks and heartbeats, the collected data might become noisy. These noises are eliminated during the pre-processing step to produce clean data that may subsequently process the feature extraction and classification. This pre-processing unit is also known as signal enhancement since it cleans the signal in BCI. Some methods are used for signal enhancement in the BCI system, and these are explained elaborately in the following subsections.

### 6.1. Independent Component Analysis (ICA)

The noises and EEG signals are isolated in ICA by treating them as distinct entities. Furthermore, the data are retained during the removal of noises. This method divides the EEG data into spatially fixed and temporally independent components. In the case of computing and noise demonstrable, the ICA shows more efficiency [256].

### 6.2. Common Average Reference (CAR)

It is most commonly employed as a basic dimensionality reduction technique. This approach decreases noise across all recorded channels, but this does not address channel-specific noise and may inject noise into an otherwise clean channel. It is a spatial filter that can be thought of as the subtraction of shared EEG activity, retaining only the idle action of each EEG particular electrode [256].

### 6.3. Adaptive Filters

The adaptive filter is a computational device for mathematical processes. It connects the adaptive filter’s input/output signals iteratively. There are filter coefficients that are self-adjusted utilizing an adaptive algorithm. It works by altering signal properties depending on the characteristics of the signals under investigation [257].

### 6.4. Principal Component Analysis (PCA)

PCA is a technique for detecting patterns in data represented by a rotation of the coordinate axes. These axes are not aligned with single time points, but they depict a signal pattern with linear combinations of sets of time points. PCA keeps the axes orthogonal while rotating them to maximize variance along the first axis. It reduces feature dimensions and aids in data classification by completing ranking. In comparison with ICA, PCA compresses separate data better whether noise is eliminated with it or not [258].

### 6.5. Surface Laplacian (SL)

SL refers to a method of displaying EEG data with a high spatial resolution. SL can be generated using any EEG recording reference scheme as their estimates are reference-free. Based on the volume conductor’s exterior shape, it is a general estimate of the current density entering or exiting the scalp through the skull, and it does not require volume conduction details. The advantage of SL is that it improves the spatial resolution of the EEG signal. However, SL seems not to demand additional operative neuroanatomy premises as it is sensitive to spline patterns and artifacts [259].

### 6.6. Signal De-Noising

Artefacts frequently corrupt EEG signals taken from brain. These artifacts must be removed from EEG data to obtain valuable information from it. The technique of eliminating sounds or artefacts from EEG signals is known as de-noising [260]. Some de-noising methods are given below:

- Wavelet de-noising and thresholding: The multi-resolution analysis is used to transfer the EEG signal to the discrete wavelet domain. The contrasting or adaptive threshold level is used to reduce particular coefficients associated with the noise signal [261]. Shorter coefficients would tend to define noise characteristics throughout time and scale in a well-matched wavelet representation. In contrast, threshold selection is one of the most critical aspects of successful wavelet de-noising. Thresholding can isolate the signal from the noise in this case; hence, thresholding approaches come in several shapes and sizes. All coefficients underneath a predetermined threshold value are set to zero in hard thresholding. Soft thresholding is a method of reducing the value of the remaining coefficients by a factor of two [262].
- Empirical mode decomposition (EMD): It is a signal analysis algorithm for multivariate signals. It breaks the signal down into a series of frequency and amplitude-regulated zero-mean signals, widely known as intrinsic mode functions (IMFs). Wavelet decomposition, which decomposes a signal into multiple numbers of Intrinsic Mode Functions (IMFs), is compared by EMD. It decomposes these IMFs using a shifting method. An IMF is a function with a single maximum between zero crossings and a mean value of zero. It produces a residue after degrading IMFs. These IMFs are sufficient to characterize a signal [263].

Most of our datasets mentioned in the previous section are a part of various BCI paradigms and follow these signal enhancement techniques as well. The motor imagery datasets represent paradigms such as sensorimotor activity or rhythms. In addition, error-related potentials datasets and datasets such as event-related potentials or visually evoke potentials signify their own BCI paradigm. Some other paradigms, such as overt attention, eye movement, miscellaneous, and emotion recognition, identify their datasets. Indeed, these paradigms become bigger in number as the measurement of different brain movements and emotions are attempted. More than 100 BCI designs are required to use signal enhancement techniques to extract features from the signal. In comparison, Reference [264] shows that 32% of BCI designs use surface Laplacian (SL) to extract features, principal component analysis (PCA) or independent component analysis (ICA) was used in 22%, and common spatial patterns (CSP) and common average referencing (CAR) techniques are used in 14% and 11%, respectively.

## 7. Feature Extraction

Now, it is necessary to understand what the features represent, their qualities, and how to use them for a BCI system to select the best appropriate classifier. A classification system’s accuracy or efficiency is primarily determined by the feature(s) of the samples to be categorized [265]; therefore, feature extraction has been crucial stage in BCI. The majority of noninvasive BCI devices use neuroimaging techniques such as MEG and MRI. However, EEG is the most widely utilized method, owing to its high temporal resolution and inexpensive cost [266]. The EEG signal feature extraction method is one of the essential components of a BCI system because of its involvement in successfully executing the classification stage at discriminating mental states. Nevertheless, the feature extraction methods based on both EEG and ECoG are discussed elaborately in the subsequent section.

### 7.1. EEG-Based Feature Extraction

Typically, BCI focuses on identifying acquired events using various neuroimage techniques, the most common of which is electroencephalography (EEG). Since its involvement in successfully executing the classification stage at discriminating mental states, the EEG signal feature extraction method is one of the essential components of a BCI system. According to [267] on EEG, three types of feature extraction are discussed in detail in the following sections. These features are the time domain, the frequency domain, and the time–frequency domain. The following subsection address the feature domains elaborately.

#### 7.1.1. Time Domain

The time–frequency domain integrates analyses in the time and frequency domains. It depicts the signal energy distribution in the Time–Frequency plane (t-f plane) [268]. When it comes to deciphering rhythmic information in EEG data, a time–frequency analysis comes in handy. EEG’s time-domain properties are straightforward to fix, but they have the disadvantage of containing non-stationary signals that alter over time. Features are usually derived using signal amplitude values in time-domain approaches that can be distorted by interference as noise during EEG recording.

- Event related potentials: Event-related potentials (ERPs) are very low voltages generated in brain regions in reaction to specific events or stimuli. They are time-locked EEG alterations that provide a safe and noninvasive way to research psychophysiological aspects of mental activities. A wide range of sensory, cognitive, or motor stimuli can trigger event-related potentials [269,270]. ERPs are useful to measure the time to process a stimulus and a response to be produced. The temporal resolution of event-related potentials is remarkable, but it has a low spatial resolution. ERPs were used by Changoluisa, V. et al. [271] to build an adaptive strategy for identifying and detecting changeable ERPs. Continuous monitoring of the curve in ERP components takes account of their temporal and spatial information. Some limitations of ERPs are that it shows poor spatial resolution, whether it is suitable with temporal resolution [272]. Furthermore, a significant drawback of ERP is the difficulty in determining where the electrical activity originates in the brain.
- Statistical features: Several statistical characteristics were employed by several scholars [273,274,275] in their research:
  − Mean absolute value:
    (1) $$MAV=\frac{1}{N}\sum_{n=1}^{N}\left|x_{n}\right|$$
  − Power:
    (2) $$P=\frac{1}{N}\sum_{n=1}^{N}{\left|x_{n}\right|}^{2}$$
  − Standard deviation:
    (3) $$SD=\sqrt{\frac{1}{N}\sum_{n=1}^{N}\left(x\left(n\right)-\mu_{n}\right)}$$
  − Root mean square (RMS):
    (4) $$\mathrm{RMS}={\left(\frac{1}{N}\sum_{i=1}^{N}x_{i}^{2}\right)}^{1/2}$$
  − Square root of amplitude (SRA):
    (5) $$\mathrm{SRA}={\left(\frac{1}{N}\sum_{i=1}^{N}\sqrt{\left|x_{i}\right|}\right)}^{2}$$
  − Skewness value (SV):
    (6) $$\mathrm{SV}=\frac{1}{N}\sum_{i=1}^{N}{\left(\frac{x_{l}-\bar{x}}{\sigma }\right)}^{3}$$
  − Kurtosis value (KV):
    (7) $$\mathrm{KV}=\frac{1}{N}\sum_{i=1}^{N}{\left(\frac{x_{l}-\bar{x}}{\sigma }\right)}^{4}$$
  where x(n) is the pre-processed EEG signal with *N* number of samples; $\mu_{n}$ refers to the meaning of the samples. Statistical features are useful at low computational cost.
- Hjorth features: Bo Hjorth introduced the Hjorth parameters in 1970 [276]; the three statistical parameters employed in time-domain signal processing are activity, mobility, and complexity. Dagdevir, E. et al. [277] proposed a motor imagery-based BCI system where the features were extracted from the dataset using the Hjorth algorithm. The Hjorth features have advantages in real-time analyses as it has a low computation cost. However, it has a statistical bias over signal parameter calculation.
- Phase lag index (PLI): The functional connectivity is determined by calculating the PLI for two pairs of channels. Since it depicts the actual interaction between sources, this index may help estimate phase synchronization in EEG time series. PLI measures the asymmetry of the distribution of phase differences between two signals. The advantage of PLI is that it is less affected by phase delays. It quantifies the nonzero phase lag between the time series of two sources, making it less vulnerable to signals. The effectiveness of functional connectivity features evaluated by phase lag index (PLI), weighted phase lag index (wPLI), and phase-locking value (PLV) on MI classification was studied by Feng, L.Z. et al. [278].

#### 7.1.2. Frequency Domain

When analyzing any signal in terms of frequency instead of just time, the frequency domain properties are considered. Any signal’s frequency domain representation displays how much of it falls inside a specific frequency range. The frequency domain properties are commonly acquired using power spectral density (PSD). The discussion about these properties is presented below in the following section.

**Figure 5:**
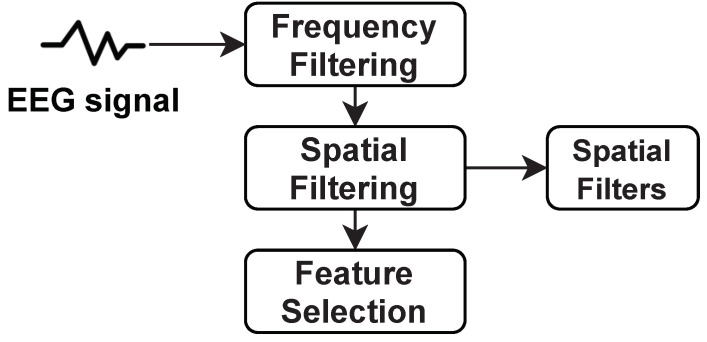
The basic structure of CSP [286].

1. Fast fourier transform (FFT): The Fourier transform is a mathematical transformation that converts any time-domain signal into its frequency domain. Discrete Fourier Transform (DFT) [279], Short Time Fourier Transform (STFT) [280,281], and Fast Fourier Transform are the most common Fourier transform utilized for EEG-based emotion identification (FFT) [282]. Djamal, E.C. et al. [283] developed a wireless device that is used to record a player’s brain activity and extracts each action using Fast Fourier Transform. FFT is faster than any other method available, allowing it to be employed in real-time applications. It is a valuable instrument for signal processing at a fixed location. A limitation of FFT is that it can convert the limited range of waveform data and the requirement to add a window weighting function to the waveform to compensate for spectral leakage.
2. Common spatial patterns (CSP): It is a spatial filtering technique usually employed in EEG and ECoG-based BCIs to extract classification-relevant data [284]. It optimizes the ratio of their variances whenever two classes of data are utilized to increase the separability of the two classes. In the case of dimensionality reduction, if a different dimension reduction phase precedes CSP, it appears to be better and has more essential generalization features. The basic structure of the CSP can be described by the Figure 5.
  In Figure 5, CSP provides spatial filters that minimize the variance of an individual class while concurrently maximizing the variance of other classes. These filters are mainly used to choose the frequency from the multichannel EEG signal. After frequency filtering, spatial filtering is performed using spatial filters that are employed to extract spatial information from the signal. Spatial information is significantly necessary to differentiate intent patterns in multichannel EEG recordings for BCI. The performance of this spatial filtering depends on the operational frequency band of EEG. Therefore, CSP is categorized as a frequency domain feature. However, CSP acts as signal enhancement while it requires no preceding excerpt or information of sub-specific bands.
3. Higher-order Spectral (HOS): Second-order signal measurements include the auto-correlation function and the power spectrum. Second-order measures operate satisfactorily if the signal resembles a Gaussian probability distribution function. However, most of the real-world signals are non-Gaussian. Therefore, Higher-Order Spectral (HOS) [285] is an extended version of the second-order measure that works well for non-Gaussian signals, when it comes into the equation. In addition, most of the physiological signals are nonlinear and non-stationary. HOS are considered favorable to detect these deviations from the signal’s linearity or stationarity. It is calculated using the Fourier Transform at various frequencies.
  (8) $$HOS=X\left(K\right)X\left(l\right)X^{*}\left(k+l\right)$$
  where X(K) is the Fourier transform of the raw EEG signal x(n) and *l* is a shifting parameter.

#### 7.1.3. Time–Frequency Domain

In the time-frequency domain, the signal is evaluated both in the time and frequency domains simultaneously. The wavelet transform is one of many advanced approaches for analyzing the time-frequency representation. There are some other widely used models for utilizing the time-frequency domain. These models are addressed with a proper explanation in the subsequent section.

1. Autoregressive model: For EEG analysis, the Autoregressive (AR) model has been frequently employed. The central premise of the autoregressive (AR) model is that the real EEG can be approximated using the AR process. With this premise, the approximation AR model’s order and parameters are set to suit the observed EEG as precisely as possible. AR produces a smooth spectrum if the model order is too low, while it produces false peaks if it is too high [287]. AR also reduces leakage and enhances frequency resolution, but choosing the model order in spectral estimation is difficult. The observational data, denoted as x(n), results from a linear system with an H(z) transfer function. Then, x(n) encounters an AR model of rank *p* in the formula [288].
  (9) $$x\left(n\right)=-\sum_{i=1}^{p}ap\left(i\right)x\left(n-i\right)+v\left(n\right)$$
  The AR parameters are ap(i), the observations are x(n) and the excitation white noise is v(n). Lastly, the most challenging part of AR EEG modeling is choosing the correct model to represent and following the changing spectrum correctly.
2. Wavelet Transform (WT): The WT technique encodes the original EEG data using wavelets, which are known as simple building blocks. It looks at unusual data patterns using variable windows with expansive windows for low frequencies and narrow windows for high frequencies. In addition, WT is considered an advanced approach as it offers a simultaneous localization in the time-frequency domain, which is a significant advantage. These wavelets can be discrete or continuous and describe the signal’s characteristics in a time-domain frequency. The Discrete Wavelet Transform (DWT) and the Continuous Wavelet Transform (CWT) are used frequently in EEG analysis [289]. DWT is now a more widely used signal processing method than CWT as CWT is very redundant. DWT decomposes any signal into approximation and detail coefficients corresponding to distinct frequency ranges maintaining the temporal information in the signal. However, most researchers try all available wavelets before choosing the optimal one that produces the best results, as selecting a mother wavelet is challenging. In wavelet-based feature extraction, the Daubechies wavelet of order 4 (db4) is the most commonly employed [290].

### 7.2. ECoG-Based Features

Electrocorticography (ECoG) generates a reliable signal through electrodes placed on the surface of the human brain, which decodes movement, vision, and speech. Decoding ECoG signal processing gives immediate patient feedback and controls a computer cursor or perhaps an exoskeleton. The ECoG signal feature extraction approach is a crucial element of the BCI system since it is involved in accomplishing the classification phase during decoding. Some of the widely used feature extraction methods are discussed below.

#### 7.2.1. Linear Filtering

It is typically employed to filter out noise in the form of signals that are not in the frequency range of the brain’s messages. Low-pass filters and high-pass filters are the two types of linear filters. This typical linear filtering is used to removed ECOG, EOG, and EMG artifacts from EEG signals. Low pass filtering is used to remove EMG artifacts, and high pass filtering is used to remove EOG artifacts [291]. These artifacts are noises produced by either physiological processes such as muscle, eye, or other biological movement or exogenous (external) sources such as machinery faults. There are three approaches for dealing with artifacts in EEG signal acquisition. Avoiding artifacts by keeping an eye on the subject’s movements and the machine’s operation. Contaminated trials are discarded due to artifact rejection. Pre-processing techniques are used to remove artifacts. The advantage of linear filtering is that signals are considered a controlled scaling of the signal’s frequency domain components. High pass filtering is used to raise the relative importance of the high-frequency components by reducing the features in the frequency domain’s center.

#### 7.2.2. Spatial Filtering

Spatial filtering is a technique for improving decoding by leveraging information about the electrode positions. The spatial filter aims to lessen the influence of spatial distortion in the raw signal; various ECoG channels are treated as coordinates for multivariate data sampling through spatial filters. The filtering transforms that coordinate system to facilitate decoding. Spatial filtering can use to minimize data dimensionality or to increase the dissimilarity of various observations. The referencing systems used during ECoG recordings are frequently utilized for preliminary spatial filtering. Equation (10) determines the spatial filter [292].

(10) $$x^{\prime }=\sum_{i}^{n}\left(x_{i}\right)*\left(w_{i}\right)$$

where $x^{\prime }$ is the spatially filtered signal, $x_{i}$ is the EEG signal from channel *i*, and $w_{i}$ is the weight of that channel. With the aid of relevant information acquired from multiple EEG channels, spatial filtering contributes to recovering the brain’s original signal. Simultaneously, it reduces dimensionality by lowering EEG channel size to smaller spatially filtered signals.

Thus far, feature extraction involves extracting new features from existing ones to minimize feature measurement costs, to improve classifier efficiency, and to improve classification accuracy. Now in the following section, the extracted feature classifiers are briefly described.

## 8. BCI Classifiers

**Figure 6:**
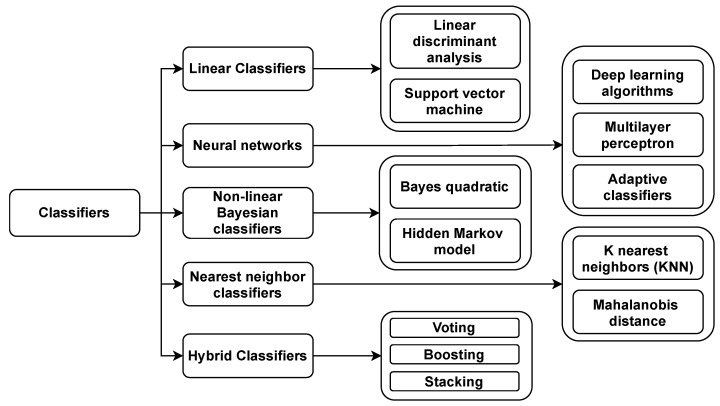
Classification of commonly used classifiers in BCI.

BCI always needs a subject to use its device, and similarly, the subject must produce several types of data to use a BCI device. In addition, to use a BCI system, the subject must develop various brain activity patterns that the system can recognize and convert into commands. To achieve this mentioned conversion, some regression or classification algorithms can be used. The classification step’s design comprises selecting one or more classification algorithms from a variety of options. In this section, some commonly known classifiers [293], which are classified in Figure 6, as well as some new classifiers [294] are described below.

### 8.1. Linear Classifiers

Linear classifiers are discriminant algorithms that discriminate classes using linear functions. It is most likely the most widely used algorithm in BCI systems. Two types of linear classifiers are used during BCI design: linear discriminant analysis (LDA) and support vector machine (SVM).

#### 8.1.1. Linear Discriminant Analysis (LDA)

The objective of Linear Discriminant Analysis is to separate data from diverse classes using a hyperplane. The side of hyperplane determinded through the category of a feature vector in a two-class problem. LDA requires that the data has a normal distribution and that both classes have the same covariance matrix. The separation hyper-plane is based on looking for a projection that maximizes the margin between the means of two classes while minimizing intraclass variance [295]. Furthermore, this classifier is straightforward to apply and generally produces excellent results and soundly implemented in various BCI system, including MI-based BCI, P300 speller, multiclass, and asynchronous BCI. The disadvantage of LDA is its linearity, which might lead to unsatisfactory results when faced with various nonlinear EEG data.

#### 8.1.2. Support Vector Machine (SVM)

A Support Vector Machine (SVM) uses a discriminant hyperplane to identify classes. The determined hyperplane in SVM is the one that maximizes the margins, i.e., the distance between both the nearest training samples. The ability to generalize is believed to improve when margins are maximized [296]. Linear SVM [297] is a type of SVM that allows for classification utilizing linear decision bounds. This classifier has been used to solve a substantial number of synchronous BCI tasks with tremendous success. The SVM classifier also works by projecting the input vector X onto a scalar value f(X), as shown in Equation (11).

(11) $$f\left(X\right)=\sum_{l=1}^{N}a_{1}y_{l}K\left(X_{l},X\right)+b$$

Gaussian SVM or RBF SVM is the term applied to the equivalent SVM. RBF and SVM have also produced remarkable outcomes in BCI applications. SVM is used to solve multiclass BCI problems that use the OVR approach, similar to LDA.

### 8.2. Neural Networks (NN)

Neural networks (NN) and linear classifiers are the two types of classifiers most usually employed in BCI systems, considering that a NN is a collection of artificial neurons that allows us to create nonlinear decision limits [298]. The multilayer perceptron (MLP) is the most extensively used NN for BCI, as described in this section. Afterward, it briefly discusses other neural network architectures utilized in BCI systems.

#### 8.2.1. Deep Learning (DL) Models

Deep learning has been widely used in BCI applications nowadays compared with machine learning technologies because most BCI applications require a high level of accuracy. Deep learning models perform better in recognizing changing signals from the brain, which changes swiftly. Some popular DL models such as CNN, GNN, RNN, and LSTM are described below:

- Convolutional Neural Network (CNN): A convolutional neural network (CNN) is an ANN intended primarily to analyze visual input used in image recognition and processing. The convolutional layer, pooling layer, and fully connected layer are the three layers that comprise CNN. Using a CNN, the input data may be reduced to instant response formations with a minimum loss, and the characteristic spatial relationships of EEG patterns can be recorded. Fatigue detection, sleep stage classification, stress detection, motor imagery data processing, and emotion recognition are among the EEG-based BCI applications using CNNs. In BCI, the CNN models are used in the input brain signals to exploit the latent semantic dependencies.
- Generative Adversarial Network (GAN): Generative adversarial networks are a recent ML technique. The GAN used two ANN models for competing to train each other simultaneously. GANs allow machines to envision and develop new images on their own. EEG-based BCI techniques recorded the signals first and then moved to the GAN techniques to regenerate the images [299]. The significant application of GAN-based BCI systems is data augmentation. Data augmentation increases the amount of training data available and allows for more complicated DL models. It can also reduce overfitting and can increase classifier accuracy and robustness. In the context of BCI, generative algorithms, including GAN, are frequently used to rebuild or generate a set of brain signal recordings to improve the training set.
- Recurrent Neural Network (RNN): RNNs’ basic form is a layer with the output linked to the input. Since it has access to the data from past time-stamps, and the architecture of an RNN layer allows for the model to store memory [300,301]. Since RNN and CNN have strong temporal and spatial feature extraction abilities in most DL approaches, it is logical to mix them for temporal and spatial feature learning. RNN can be considered a more powerful version of hidden Markov models (HMM), which classifies EEG correctly [302]. LSTM is a kind of RNN with a unique architecture that allows it to acquire long-term dependencies despite the difficulties that RNNs confront. It contains a discrete memory cell, a type of node. To manage the flow of data, LSTM employs an architecture with a series of “gates”. When it comes to modeling time series of tasks such as writing and voice recognition, RNN and LSTM have been proven to be effective [303].

#### 8.2.2. Multilayer Perceptron (MLP)

An Multilayer Perceptron (MLP) [304] comprises multiple layers of neurons along with an input layer, one or more hidden layers, and an output layer. The input of each neuron is linked to the output of the neurons in the preceding layer. Meanwhile, the output layer neurons evaluate the classification of the input feature vector. MLP and neural networks can approximate, meaning they can compare continuous functions if they have sufficient neurons and layers. The challenging factor behind MLPs is that they are susceptible to over-training, particularly containing noisy and non-stationary data. As a result, significant selection and regularization of the architecture are necessary. Perceptron is a multilayer with no hidden layers comparable with LDA. It has been used in BCI applications on occasion [293]. Sunny, M.S.H. et al. [305] used Multilayer Perceptron (MLP) to distinguish distinct frequency bands from EEG signals to extract features more effectively.

#### 8.2.3. Adaptive Classifiers

As new EEG data become accessible, adaptive classifiers’ parameters, such as the weights allocated to each feature in a linear discriminant hyperplane, are gradually re-estimated and updated. Adaptive classifiers can use supervised and unsupervised adaptation, that is, with or without knowledge of the input data’s real class labels. The true class labels of the receiving EEG signals are obtained using supervised adaptation. The classifier is either reassigned on the existing training data, enhanced with these updated, labeled incoming data, or updated solely on this new data. Supervised user testing is essential for supervised BCI adaptation. The label of the receiving EEG data is vague with unsupervised adaptation. As a result, unsupervised adaptation is based on class-unspecific adaptation, such as updating the generalized classes EEG data mean or a co-variance matrix in the classifier model or estimating the data class labels for additional training [306].

### 8.3. Nonlinear Bayesian Classifiers

This section discusses the Bayes quadratic and hidden Markov models (HMM), two Bayesian classifiers used in BCI. Although Bayesian graphical networks (BGN) have been used for BCI, they are not covered here since they are not widely used [307].

#### 8.3.1. Bayes Quadratic

The objective of Bayesian classification is to provide the highest probability class to a feature vector. The Bayes rule is often used to calculate the a posteriori probability of a feature vector assigned to a single class. The class of this feature vector can be calculated by using the MAP (maximum a posteriori) rule with these probabilities. The Bayes quadratic assumption is that the data have a distinct normal distribution. The result is quadratic decision boundaries that justify the classifier’s name [308]. Although this classifier is not extensively utilized for BCI, it has been successfully used to classify motor imagery and mental tasks.

#### 8.3.2. Hidden Markov Model

A Bayesian classifier that generates a nonlinear cost function is known as a Hidden Markov Model (HMM). An HMM is a statistical algorithm that calculates the chances of seeing a given set of feature variables [309]. These statistical probabilities from HMM are generally Gaussian Mixture Models (GMM) in case of BCI [310]. HMM may be used to categorize temporal patterns of BCI characteristics (Obermaier, B. et al. [302]), even raw EEG data, since the EEG elements required to control BCI have particular time sequences. Although HMM is not widely used in the BCI world, this research demonstrated that they could be helpful to classification on BCI systems such as EEG signals [311].

### 8.4. Nearest Neighbor Classifiers

In this section, some classifiers with distance vectors are described. Classifiers such as K nearest neighbors (KNN) and Mahalanobis distance are common among them as they are nonlinear discriminative classifiers [312].

#### 8.4.1. K Nearest Neighbors

K nearest neighbor method aims to identify the dominant class amongst an unseen point within the dataset habituated for training. Nearest neighbors are typically estimated using a metric that has some intervals during the signal acquisition of BCI. KNN can construct nonlinear decision boundaries by evaluating any function with enough training data with an inflated k value. The usability of KNN algorithms is less in the BCI field as their condescending sensitivity hampers the capacity, which causes them to fail in multiple BCI research. KNN is efficient in BCI systems with some feature vectors, but low power can cause failure in BCI research [313].

#### 8.4.2. Mahalanobis Distance

For each prototype of class *c*, Mahalanobis distance-based classifiers [314] assume a Gaussian distribution N(c,Mc). Subsequently, using the Mahalanobis distance dc, a feature vector *x* is allocated to the class that corresponds to the closest prototype (*x*).

(12) $$d_{c}\left(x\right)=\sqrt{\left(x-\mu_{c}\right)M_{c}^{-1}{\left(x-\mu_{c}\right)}^{T}}$$

This results in a basic yet reliable classifier; it has been shown to work in multiclass and asynchronous BCI systems. Considering its excellent results, it is still rarely mentioned in BCI literature [315].

### 8.5. Hybrid

In several BCI papers, classification is implemented with a single classifier. Furthermore, a current tendency is to combine many classifiers in various ways [316]. The following are indeed the classifier combination strategies utilized in BCI systems:

#### 8.5.1. Boosting

Boosting is the process of using multiple classifiers in a cascade, and each focused on the errors made by the one before it. It can combine numerous weak classifiers to form a powerful one; thereforem it is unlikely to overtrain. Moreover, it is susceptible to mislabeling, illustrating why it failed in one BCI trial [293].

#### 8.5.2. Voting

Multiple classifiers are employed for voting, each of which allocates the input feature vector to a class. The majority class becomes the final class. In BCI systems, voting is the most preferred process of combining classifiers due to its simplicity and efficiency [293].

#### 8.5.3. Stacking

Stacking is the process of utilizing multiple classifiers to categorize the input feature vector. Level-0 classifiers are what it is named. Each one of these classifiers’ output would then feed into a “meta-classifier” (or “level-1 classifier”), which makes a final decision [293].

Aforementioned in this section, some other classifiers are utilized in the recent BCI research. Since 2016 transfer learning is used for using MI classification tasks [317]. Some ground-breaking architectures are established in recent years, such as EEG-inception, an end-to-end Neural network [318], cluster decomposing, and multi-object optimization-based-ensemble learning framework [319]; RFNet is a fusion network that learns from attention weights and used in embedding-specific features for decision making [179].

Now, a better understanding of the performance of commonly known classifiers with some popular datasets are given in Table 9.

## 9. Evaluation Measurement

To evaluate the performance of BCI systems, researchers employed several evaluation metrics. The most common is accuracy, commonly known as error rate. Although accuracy is not always an acceptable criterion due to specific rigorous requirements, various evaluation criteria have been offered. An overview of BCI research evaluation criteria is provided below.

### 9.1. Generally Used Evaluation Metrics

In this section, we sorted the most commonly used evaluation metrics for measuring the BCI system performances. The evaluation measures are explained carefully in the following subsections.

#### 9.1.1. The Confusion Matrix

The confusion matrix represents the relationship between the actual class’s user-intentioned output classes and the actual predicted class. True positives rate (TPR), False negative rate (FNR), False positives rate (FPR), Positive predictive value (PPV), and negative predictive value (PPV) are used to describe sensitivity or recall, specificity, (1-specificity), precision, etc. [325].

#### 9.1.2. Classification Accuracy and Error Rate

Classification accuracy is one of the important metrics in BCI systems; this study evaluates performance using classification accuracy as well as sensitivity and specificity. This measure determines how frequently the BCI makes a right pick or what proportion of all selections are accurate. It is the most obvious indicator of BCI accomplishment, implying that it increase in a linear fashion with decision time, so it takes a long time. The following is the mathematical formula for calculating accuracy:

(13) $$\mathrm{Classification}\;\mathrm{accuracy}=\frac{\mathrm{Correctly}\;\mathrm{classified}\;\mathrm{test}\;\mathrm{trials}}{\mathrm{Total}\;\mathrm{test}\;\mathrm{triols}}\times 100$$

#### 9.1.3. Information Transfer Rate

Shannon [326] proposed the Information Transfer Rate (ITR) as the rate that makes up both of these metrics. This rate represents the quantity of data that may pass through the system in one unit of time. In [327], the information transmission rate in bits per minute (bits/min) and accuracy (ACC) in percentage (%) were used to evaluate performance. They made demographic data (age and gender) as well as the performance outcomes of 10 participants, and the ITR was computed using the Formula (14), which is as follows:

(14) $$B_{t}=\log_{2}N+p\log_{2}p+\left(1-p\right)\log_{2}\left[\frac{1-p}{N-1}\right],$$

where *N* is the number of targets and *p* is the classification accuracy (ACC). Based on four cursor movements and the choose command, this resulted in a *N* of 5. Bits per trial were used to compute $B_{t}$.

According to ITR [328] also has some important parameters that are used to evaluate BCI. A description of them is given below:

1. Target detection accuracy: The accuracy of target identification may be enhanced by increasing the Signal-to-Noise Ratio (SNR) and the separability of several classes. Several techniques, such as trial averaging, spatial filtering, and eliciting increased task-related EEG signals, are employed in the preprocessing step to reduce the SNR. Many applications utilize trail averaging across topics to improve the performance of a single BCI. These mental states may be used to lower the SNR [53].
2. Number of classes: The number of classes is raised and more sophisticated applications are built with a high ITR. TDMA, FDMA, and CDMA are among the stimulus coding techniques that have been adopted for BCI systems [243,329]. P300, for example, uses TDMA to code the target stimulus. In VEP-based BCI systems, FDMA and CDMA have been used.
3. Target detection time: The detection time is when a user first expresses their purpose and when the system makes a judgment. One of the goals of BCI systems is to improve the ITR by reducing target detection time. Adaptive techniques, such as the “dynamic halting” method, might be used to minimize the target detection time [330].

#### 9.1.4. Cohen’s Kappa Coefficient

Cohen’s Kappa measures the agreement between two observers; it measures the contract between the proper output and the command of BCI domain in a BCI-based AAC system. Cohen’s kappa coefficient resolves many of the accuracy measure’s objections [331]. The general agreement $p_{0}=ACC$, which is equivalent to the classification accuracy and the chance agreement $p_{e}$, with $n_{i}$ and $n_{i_{i}}$ being the column $i_{t}h$ and row $i_{t}h$, correspondingly, are used to calculate *K*.

(15) $$p_{e}=\frac{\sum_{i=1}^{M}n_{ii}n_{i:}}{N^{2}}$$

where posteriori and priori probability are n:i, ni: respectively. The estimated kappa Coefficient *K* and standard error e(K) are acquired by

(16) $$\kappa =\frac{p_{0}-p_{e}}{1-p_{e}}$$

When there is no correlation between the expected and actual classes, the kappa coefficient becomes zero. A perfect categorization is indicated by a kappa coefficient of 1. If the Kappa value is less than zero, the classifier offers an alternative assignment for the output and actual classes [332].

(17) $$\sigma_{e}\left(\kappa \right)=\frac{\sqrt{\left(p_{0}+p_{e}^{2}-\sum_{i=1}^{M}\left[n_{:i}n_{i:}\left(n_{:i}+n_{i:}\right)\right]/N^{3}\right)}}{\left(1-p_{e}\right)\sqrt{N}}$$

### 9.2. Continuous BCI System Evaluation

Continuous BCI performance was measured using a variety of parameters. Different measures may be even more appropriate depending on whether the study is conducted online or offline. The section goes through some of the most commonly used metrics in this field, including the correlation coefficient, accuracy, and Fitts’s Law [333].

#### 9.2.1. Correlation Coefficient

The correlation coefficient could be a useful statistic for determining whether an intracortical implant receives task-relevant neurons. There are two essential stipulations: one is scale-invariant, which implies that the cursor might miss the mark substantially while still generating high values if the sign of the actual and anticipated movements coincide [334]; the other is that a decoder can yield a high value if it simply generates a signal that fluctuates with the repetitions [333].

#### 9.2.2. Accuracy

Task characteristics such as target size and dwell time have a significant impact on accuracy. As a result, it is more of a sign that the task was is good enough for the subject and modality than a performance measure [333].

#### 9.2.3. Fitts’s Law

Fitts’s law asserts that the time taken for a person to move a mouse cursor to a targeted object of the target’s distance is divided by its size. The longer it takes, the greater the distance and the narrower the target [335,336]. Fitts’s law requires using a method to calculate the “index of difficulty” of a particular change.

### 9.3. User-Centric BCI System Evaluation

Users are an essential element of the BCI product life cycle. Their interactions and experiences influence whether BCI systems are acceptable and viable. The four criteria or User Experience (UX) factors are used to evaluate user-centric BCI systems. These are usability, affects, ergonomics, and quality of life, shown below in the following subsection.

#### 9.3.1. Usability

The amount that can be utilized to fulfill specific objectives with effectiveness, efficiency, learnability, and satisfaction in a given context is referred to as usability [337]. In usability measure, we can include four metrics, such as,

1. Effectiveness or accuracy: It depicts the overall accuracy of the BCI system as experienced from the end user’s perspective [333].
2. Efficiency or information transfer rate: It refers to the speed and timing at which a task is accomplished. Therefore, it depicts the overall BCI system’s speed, throughput, and latency seen through the eyes of the end user’s perspective [333].
3. Learnability: The BCI system can make users feel as if they can use the product effectively and quickly learn additional features. Both the end-user and the provider are affected by learnability [338].
4. Satisfaction: It is based on participants’ reactions to actual feelings while using BCI systems, showing the user’s favorable attitude regarding utilizing the system. To measure satisfaction, we can use rating scales or qualitative methods [333].

#### 9.3.2. Affect

Regarding BCIs, it might refer to how comfortable the system is, particularly for long periods, and how pleasant or uncomfortable the stimuli are to them. EEG event-related possibilities, spectral characteristics, galvanic skin responses, or heart rates could be used to quantitatively monitor user’s exhaustion, valence, and arousal levels [339].

#### 9.3.3. Ergonomics

Ergonomics studies are the study of how people interact with their environments. The load on the user’s memory is represented by the cognitive task load, a multidimensional entity. In addition, physiological markers including eye movement, EEG, ERP, and spectral characteristics could also be employed to evaluate cognitive stress objectively [340].

#### 9.3.4. Quality of Life

It expresses the user’s overall perception of the system’s utility and acceptance and its influence on their well-being. The Return on Investment (ROI) is an economic measure of the perceived benefit derived from it. The overall quality of experience is a measure of how satisfied a user is with their expertise [333].

Other assessment methods, such as Mutual Information, Written symbol rate (WSR), and Practical bit rate (PBR), are utilized to a lesser extent.

## 10. Limitations and Challenges

The brain-computer interface is advancing towards a more dynamic and accurate solution of the connection between brain and machine. Still, few factors are resisting achieving the ultimate goal. Therefore, we analyzed a few core research on BCI in this section and found the limitations exhibited in Table 10. Then, we demonstrated the significant challenges of the BCI domain.

The challenges and difficulties of the BCI domain are divided into three categories: challenges based on usability, technical challenges, and ethical challenges. The rest of the section briefly explains these challenges.

### 10.1. Based on Usability

This section describes the challenges that users have in accepting BCI technology [350]. They include concerns relating to the requisite training for class discrimination.

#### 10.1.1. Training Time

Usually, training a user, either leading the user through the procedure or the total quantity of the documented manual, takes time. The majority of the time, the user also requests the system to be simpler to use. The users often despise a complicated system that is difficult to manage. It is a challenging effort to create such a sophisticated, user-friendly system [351].

#### 10.1.2. Fatigue

The majority of present BCIs generate a lot of fatigue since they need a lot of concentration, focus, and awareness to a rapid and intermittent input. In addition to the annoyance of weariness of electrodes, BCI may fail to operate because the user cannot maintain a sufficient degree of focus. As in BCI, mental activity is continually monitored and the user’s attention point alters the input. The concentration necessary for stimuli results in a combination of input and output [352,353]. Rather than relaxing, the user must concentrate on a single point as an input and then look at the outcome. At some point, the interaction has a forced quality to it, rather than the natural quality that would be there if the user could choose whatever part of the visual output to focus on [6].

#### 10.1.3. Mobility to Users

Across most situations, users are not allowed to move around or to have mobility in BCIs. During the test application, users must stay motionless and quiet, ideally sitting down. However, in a real-world setting, a user may need to utilize BCI while walking down the street, for example, to manage a smartphone. Additionally, BCIs cannot ensure user comfort. Usually, the EEG headset is not lightweight and easy to carry, which hampers the user experience.

#### 10.1.4. Psychophysiological and Neurological Challenges

Emotional and mental mechanisms, cognition-related neurophysiology, and neurological variables, such as functionality and architecture, play vital roles in BCI performance, resulting in significant intra- and inter-individual heterogeneity. Immediate brain dynamics are influenced by psychological elements such as attention; memory load; weariness; conflicting cognitive functions; and users’ specific characteristics such as lifestyle, gender, and age. Participants with weaker empathy engage less emotionally in a P300-BCI paradigm and generate larger P300 wave amplitudes than someone with greater empathy involvement [354].

### 10.2. Technical Challenges

Non-linearity, non-stationarity, and noise as well as limited training sets and the accompanying dimensionality curse are difficulties relating to the recorded electrophysiological characteristics of brain impulses.

#### 10.2.1. Non-Linearity

The brain is a very complex nonlinear system in which chaotic neuronal ensemble activity may be seen. Nonlinear dynamic techniques can thus better describe EEG data than linear ones.

#### 10.2.2. Non-Stationarity

The non-stationarity of electrophysiological brain signals to recognize human recognition is a significant challenge in developing a BCI system. It results in a constant shift in the signals utilized with time, either between or within transition time. EEG signal variability can be influenced by the mental and emotional state backdrop across sessions. In addition, various emotional states such as sadness, happiness, anxiety, and fear can vary on daily basis that reflects non-stationarity [355]. Noise is also a significant contribution to the non-stationarity problems that BCI technology faces. Noises and other external interferences are always present in raw EEG data of emotion recognition that is most robust [356]. It comprises undesired signals generated by changes in electrode location as well as noise from the surroundings [357].

#### 10.2.3. Transfer Rate of Signals

In BCIs, the system must continuously adjust to the signals of the user. This modification must be made quickly and precisely. Current BCIs have an extremely slow information transfer rate, taking almost two minutes to “digitalize” a single phrase, for example. Furthermore, BCI accuracy does not always reach a desirable level, particularly in visual stimulus-based BCI. Actions must sometimes be repeated or undone, producing pain or even dissatisfaction in using interactive systems using this type of interface [358].

#### 10.2.4. Signal Processing

Recently, a variety of decoding techniques, signal processing algorithms, and classification algorithms have been studied. Despite this, the information retrieved from EEG waves does not have a high enough signal-to-noise ratio to operate a device with some extent of liberty, such as a prosthetic limb. Algorithms that are more resilient, accurate, and quick are required to control BCI.

#### 10.2.5. Training Sets

In BCI, the training process is mainly impacted by usability concerns, but training sets are tiny in most cases. Although the subjects find the training sessions time-consuming and challenging, they give the user the required expertise to interact with the system and to learn to manage their neurophysiological signals. As a result, balancing the technological complexity of decoding the user’s brain activity with the level of training required for the proper functioning of the interfaces is a crucial issue in building a BCI [359].

#### 10.2.6. Lack of Data Analysis Method

The classifiers should be evaluated online since every BCI implementation is in an online situation. Additionally, it should be validated to ensure that it has low complexity and can be calibrated rapidly in real-time. Domain adaptation and transfer learning could be an acceptable solution for developing calibration-free BCIs, where even the integration of unique feature sets, such as covariance matrices with domain adaptation algorithms, can strengthen the invariance performance of BCIs.

#### 10.2.7. Performance Evaluation Metrics

A variety of performance evaluation measures are used to evaluate BCI systems. However, when different evaluation metrics are used to assess BCI systems, it is nearly impossible to compare systems. As a result, the BCI research community should establish a uniform and systematic approach to quantify a particular BCI application or a particular metric. For example, to test the efficiency of a BCI wheelchair control, the number of control commands, categories of control commands, total distance, time consumed, the number of collisions, classification accuracy, and the average success rate need to be evaluated, among other factors [360].

#### 10.2.8. Low ITR of BCI Systems

The information transfer rate is one of the extensively used processes for the performance evaluation metrics of BCI systems. The number of classes, target detection accuracy, and target detection time are all factors of this rate. By increasing the Signal-to-Noise Ratio (SNR), it can improve the target detection accuracy [53,328]. Several techniques are typically used for the preprocessing phase to optimize the SNR. When a high ITR has been attained, more complicated applications can be created by expanding the number of classes available. CDMA, TDMA, and FDMA [243,361] are only a few of the stimulus coding schemes that have already been developed for BCI systems. TDMA was used with P300 to code the required stimuli, while CDMA and FDMA have been used with BCIs that interact with VEP. Furthermore, the essential aspect of BCIs is reducing the target recognition period, which helps to increase the ITR. Adaptive techniques, such as “dynamic stopping”, could be an effective option for accomplishing this.

#### 10.2.9. Specifically Allocated Lab for BCI Technology

Most of the BCI systems are trialed in a supervised lab rather than in the actual surroundings of the users. When designing a BCI system, it is essential to think about the environment in which the technology may be used. It is critical to thoroughly investigate the system’s requirements, environmental factors, circumstances, and target users mostly during the system design phase.

### 10.3. Ethical Challenges

There are many thoughts surrounding the ethical issues behind BCI as it considers physical, psychological, and social factors. In biological factors, BCI always finds a human body to identify signals that must be acquainted with electrodes. As humans need to wear these electrodes, it is always risky for them and can harm the human body to some worse extent. BCI also requires strict maintenance of the human body during signal acquisition, so the subject must sit for a long time in his place. Adding to that, a user or participant must act what the electrodes need, so they cannot do anything willingly. This fact can have a substantial impact on the human body.

## 11. Conclusions

The brain-computer interface is a communication method that joins the wired brain and external applications and devices directly. The BCI domain includes investigating, assisting, augmenting, and experimenting with brain signal activities. Due to transatlantic documentation, low-cost amplifiers, greater temporal resolution, and superior signal analysis methods, BCI technologies are available to researchers in diverse domains. Moreover, It is an interdisciplinary area that allows for biology, engineering, computer science, and applied mathematics research. However, an architectural and constructive investigation of the brain–computer interface is exhibited in this article. It is aimed at novices who would like to learn about the current state of BCI systems and methodologies. The fundamental principles of BCI techniques are discussed elaborately. It describes the architectural perspectives of certain unique taxons and gives a taxonomy of BCI systems. The paper also covered feature extraction, classification, evaluation procedures, and techniques as the research continues. It presents a summary of the present methods for creating various types of BCI systems. The study looks into the different types of datasets that are available for BCI systems as well. The article also explains the challenges and limitations of the described BCI systems, along with possible solutions. Lastly, BCI technology advancement is accomplished in four stages: primary scientific development, preclinical experimentation, clinical investigation, and commercialization. At present, most of the BCI techniques are in the preclinical and clinical phases. The combined efforts of scientific researchers and the tech industries are needed to avail the benefit of this great domain to ordinary people through commercialization.

## Figures and Tables

**Table 1:** A summary of recent surveys/reviews on various BCI technologies, signals, algorithms, classifiers, etc.

Ref.	Purposes	Challenges
[6]	Advantages, disadvantages, decoding algorithms, and classification methods of EEG-based BCI paradigm are evaluated.	Training time and fatigue, signal processing, and novel decoders, shared control to supervisory control in closed-loop.
[7]	A comprehensive review on the structure of the brain and on the phases, signal extraction methods, and classifiers of BCI	Human-generated thoughts are non-stationary, and generated signals are nonlinear.
[8]	A systematic review on the challenges in BCI and current studies on BCI games using EEG devices	Biased within the process of search and classification.
[9]	A well-structured review on sensors used on BCI applications that can detect patterns of the brain	The sensors are placed in the human brain when neurosurgery is needed, which is a precarious process.
[10]	A brief review on standard invasive and noninvasive techniques of BCI, and on existing features and classifiers	To build brain signal capture systems with low-density electrodes and higher resolution.
[11]	This paper briefly describes the application of BCI and neurofeedback related to haptic technologies	This study only covers a small domain of BCI (haptic technology)
[12]	This survey mainly focuses on identifying emotion with EEG-based BCI, with a brief discussion on feature extraction, selection, and classifiers	There are no real-life event datasets, and the literature could not sense the mixed feelings simultaneously.
[13]	This paper refers to applying only noninvasive techniques on BCI and profound learning-related BCI studies	This study exclusively covers noninvasive brain signals.
[14]	This review focused on popular techniques such as deep learning models and advances in signal sensing technologies	Popular feature extraction processes, methods, and classifiers are not mentioned or reviewed.

**Table 2:** A table of different types of motor imagery datasets of BCI.

Dataset Name	Subject (S)/Electrodes (E)/Channels (C)	Used in
Left or Right Hand MI [70]	S: 52	[71,72,73,74,75]
Motor Movement or Imagery Dataset	S: 109 E: 64	[76,77,78,79]
Grasp and Lift EEG [80]	S: 12	[81,82,83,84,85]
SCP data of Motor-Imagery [86]	S: 13 Recordings: 60 h	[87,88,89,90,91,92]
BCI Competition III [93]	S: 3 C: 60	[94,95,96]
BCI Competition IV-1	S: 7 C: 64	[97,98,99,100,101]
BCI Competition IV-2a	S: 9 E: 22	[102,103,104,105,106]
BCI Competition IV-2b	S: 9 E: 3	[107,108,109,110,111,112]
High-Gamma Dataset [113]	S: 14 E: 128	[114,115,116,117,118,119,120]
Left/Right Hand 1D/2D movements	S: one E: 19	[86,121,122,123]
Imagination of Right-hand Thumb Movement [124]	S: one E: 8	[83,125,126,127,128]
Mental-Imagery Dataset	S: 13	[129,130,131,132,133,134,135]

**Table 3:** A table of different types of Error-Related Potentials (ErrP) dataset of BCI.

Dataset Name	Subject (S)/Electrodes (E)/Channels (C)	Used in
BCI–NER Challenge [136]	S: 26 C: 56	[137]
ErrP in a target selection task	S: E: 64	[138,139,140,141,142,143,144]
ErrPs during continuous feedback [145]	S: 10 E: 28	[146,147,148]

**Table 4:** A table of different types emotion recognition dataset of BCI.

Dataset Name	Subject (S)/Electrodes (E)/Channels (C)	Used in
DEAP [149]	S: 32 C: 32	[150,151,152,153,154,155,156,157]
Enterface’06 [158]	S: 5 C: 54	NA
HeadIT	S: 31	[159]
NeuroMarketing [160]	S: 25 E: 14	[161,162]
SEED [163]	S: 15 C: 62	[12,164,165,166,167,168,169]
SEED-IV	S: 15 C: 62	[170,171,172,173,174,175]
SEED-VIG [176]	E: 18	[137,177,178,179]
HCI-Tagging	S: 30	[180,181,182,183,184,185,186]
Regulation of Arousal [187]	S: 18	[52,130,188,189,190]
EEG Alpha Waves [191]	S: 20	[192]

**Table 5:** A table of different types of miscellaneous datasets.

Dataset Name	Subject (S)/Electrodes (E)/Channels (C)	Used in
MNIST Brain Digits	S: Single Recordings: 2 s	[193,194]
Imagenet Brain	S: Single Recordings: 3 s	[195,196,197,198,199,200]
Working Memory [201]	S: 15 E: 64	[202,203,204,205]
Deep Sleep Slow Oscillation [201]	R: 10s	[206]
Genetic Predisposition to Alcoholism	S: 120 E: 64	[124,207,208,209,210,211,212]
Confusion during MOOC [213]	S:10	[214,215]

**Table 6:** A table of different types of eye-blink or movement datasets in BCI.

Dataset Name	Subject (S)/Electrodes (E)/Channels (C)	Used in
Voluntary-Involuntary Eye-Blinks [216]	S: 20 E: 14	[217]
EEG-eye state [124]	Recordings: 117 s	[218,219,220,221]
EEG-IO [222]	S: 20 Blinks: 25	[222,223]
Eye blinks and movements [222]	S: 12	[222,224]
Eye State Prediction [225]	S: Single Recordings: 117 s	[130,218,219,226,227,228]

**Table 7:** A table of different types Event-Related Potential (ERP) datasets in BCI. These datasets are collected from [229].

Dataset Name	Subject (S)/Electrodes (E)/Channels (C)	Used in
Target Versus Non-Target (2012)	S: 25 E: 16	NA
Target Versus Non-Target (2013)	S: 24 E: 16	[230]
Target Versus Non-Target (2014)	S: 71 E: 16	[231]
Target Versus Non-Target (2015)	S: 50 E: 32	[232,233,234]
Impedance Data	S: 12	[86,94,235,236,237,238]
Face vs. House Discrimination [239]	S: 7	[240,241]

**Table 8:** A table of different types of Visually Evoked Potential (VEP) datasets in BCI. These datasets are collected from [229].

Dataset Name	Subject (S)/Electrodes (E)/Channels (C)	Used in
c-VEP BCI	S: 9 C: 32	[242,243,244]
c-VEP BCI with dry electrodes	S: 9 C: 15	[243,245,246,247,248]
SSVEP	S: 30 E: 14	[249,250,251,252,253]
Synchronized Brainwave Dataset	Video stimulus	[254,255]

**Table 9:** Comparison of classifiers based on popular datasets and features.

Ref.	Dataset	Feature	Classifier	Accuracy
[102]	BCI competition IV-2b	CWT	CNN	Morlet- 78.93%, Bump-77.25%
[320]	BCI competition III	CSP	SVM	Evolved Filters: Subject 1—77.96%, Subject 2—75.11%, Subject 3—57.76%
[321]	BCI competition III	WT	SVM	85.54%
[321]	BCI competition III	WT	NN	82.43%
[322]	BCI competition III	WT	LDA	MisClassification Rate: 0.1286
[323]	BCI competition III	WT	CNN	86.20%
[324]	BCI competition IV-2a	Single Channel CSP	KNN	62.2 ± 0.4%
[324]	BCI competition IV-2a	Single Channel CSP	MLP	63.5 ± 0.4%
[324]	BCI competition IV-2a	Single Channel CSP	SVM	63.3 ± 0.4%
[324]	BCI competition IV-2a	Single Channel CSP	LDA	61.8 ± 0.4%

**Table 10:** A summary of some research papers proposing new methods of BCI.

Model	Novelty	Feature Extraction	Architecture	Limitations
P300, ERN, MRCP, SMR [200]	Compact Convolutional neural network for EEG based BCI	Band pass filtering	EEGNet	The proposed approaches only work effectively when the feature is accustomed to before.
WOLA [254]	Dynamic filtering of EEG signals	CSP	Embedded-BCI (EBCI) system	This model is not updated yet for eye blinking or muscle activities.
xDAWN [255]	Enhance P300 evoked potentials	Spatial Filtering	P300 speller BCI paradigm	There is room for improvization and enhancements.
SSVEP, P300 [341]	BCI-based healthcare control system	P300 detector Kernel (FDA+ SSVEP)	Self- paced P300 healthcare system with SSVEP	SSVEP stimulation paradigm can be used to enhance accuracy.
LSTM, pCNN, RCNN [342]	Online decoding of motor imagery movements using DL models	CSP, log-BP features	Classify Motor Imagery movements	The data used in proposed models are limited.
MDRM and TSLDA [343]	Classification framework for BCI-based motor imagery	Spatial filtering	MI-based BCI classification using Riemannian framework	Computational costs are faced while implementing this proposed framework.
SVM [344]	Fatigue detection system	FFT	Train driver Vigilance detection	NA
Gaussian, polynomial kernel [345]	MKELM-based method for motor imagery EEG classification	CSP	MKELM-based method for BCI	Improvement of accuracy and extension of the framework is needed.
Bimodal NIRS-EEG approach [346]	Bimodal BCI using EEG and NIRS	Low pass filter and Savitzky–Golay (SG)	SSVEP paradigm	Only used in EEG and fNIRS channels.
P300-BCI classification using CNN [347]	Detection of P300 waves	Spatial filters with CNN	NN architecture	Variability over subjects, determining key layers
Unified ELM and SB learning [348]	Sparse Bayesian ELM (SBELM)-based algorithm	CSP method	SBELM for Motor Imagery-related EEG classification	Multiband optimization can increase the accuracy.
Extended Kalman adaptive LDA [349]	Online training for controlling a simulated robot	LDA classifiers	Online self-paced event detection system	Limited to two classes and does not extend to multiple classes.

## Data Availability

There is no statement regarding the data.
